# Supplementary material for: Resting vagal tone, alpha amylase and cortisol levels in women with eating disorders before and after psychotherapy
Source: PLoS One. 2025 Sep 8;20(9):e0329573. doi: 10.1371/journal.pone.0329573 (PMC12416730; doi:10.1371/journal.pone.0329573)
Supplement: Attachment — Submitted filename: Response to Reviewers_sm_300625.docx [file pone.0329573.s002.docx]

Fribourg, June 30, 2025.

To: Editors of PLOS One

Concerning Resubmission of the Manuscript: *“*Resting vagal tone, alpha amylase and cortisol levels in Women with Eating Disorders before and after psychotherapy*”*

Dear Editor,

We would like to thank you for the possibility to resubmit our manuscript *“Resting vagal tone, alpha amylase and cortisol levels in Women with Eating Disorders before and after psychotherapy”.* The comments of the reviewers were very helpful, and we hope that the answers to the reviewers and the changes in the manuscript are satisfactory. The study was approved by a local ethics committee and conforms to the principles expressed in the Declaration of Helsinki. Written informed consent was obtained from all participants. This paper reports original, unpublished work that is not considered for publication elsewhere. We declare to have no potential conflict of interest, financially or otherwise. The present research was supported by grants from several funding agencies (SNSF 149416 and 170503, DFG SCHN 415/4, SANS 22 12, Research Found University of Fribourg: 419), but the funding agencies were not involved in our study design, data collection and analysis, decision to publish, or preparation of the manuscript.

Please let us know if we can be of any further assistance: [simone.munsch@unifr.ch](mailto:simone.munsch@unifr.ch); [nadine.messerli-burgy@unil.ch](mailto:nadine.messerli-burgy@unil.ch)

On behalf of all authors,

Shared first authors:

Dr. Simone Munsch

CH-University of Fribourg

[simone.munsch@unifr.ch](mailto:simone.munsch@unifr.ch)

Dr. Nadine Messerli

CH-University of Lausanne

[nadine.messerli-burgy@unil.ch](mailto:nadine.messerli-burgy@unil.ch)

**Point-by-point reply**

**ACADEMIC EDITOR:**

***Editor****: Thank you for submitting your manuscript to PLOS ONE. The study addresses a highly relevant topic, particularly the physiological impact of psychotherapy in women with eating disorders—an area of growing clinical and theoretical interest. The longitudinal design, the inclusion of multiple stress-related biomarkers, and the multicenter approach are all notable strengths.*

*While the study is ambitious and methodologically solid in many respects, there are conceptual and analytical issues that need to be addressed to strengthen its scientific contribution.*

**Response**: We thank the reviewer for this overall positive evaluation and agree that we need to rule out uncertainty regarding the conceptual approach to our research questions and have included most of the suggestions of the editor and the reviewers.

***Editor:*** *Most importantly, the results—although clearly reported—are modest in terms of post-treatment physiological changes. The interpretation of these findings, particularly regarding cortisol and alpha-amylase, would benefit from further critical reflection. The discussion should explore more thoroughly what these null or limited effects imply for the clinical utility of such biomarkers. Likewise, the observed decrease in vagal tone in AN/BN groups is presented as a sign of normalization, but this interpretation requires more cautious framing and stronger theoretical grounding.*

**Response**: We agree with the editor's summary. We discuss the meaning of our findings in more detail. It says now on p.23:

Contrary to our expectations, sC levels decreased less in the clinical groups (AN, BN and mixed mental disorders) during therapy than during the waiting period in healthy women, whereas, as expected, the decrease of sAA was comparable between all clinical groups and the healthy sample. These findings might point to the long-lasting effect of ED pathologies on the physiological stress system even after weight normalization (Holsen et al., 2014), which are probably not reversed after a treatment duration of three months. Such treatment-resistant HPA activation patterns have also been reported by Het and colleagues (Het et al., 2020). They investigated a small sample of 13 young women with ED in comparison with a non-clinical sample and found at discharge from an in-patient treatment (at an average of 8.47 weeks SEM ± 1.8 (range: 2–58 weeks) after the first assessment) unchanged cortisol responses to a lab-based stress task. In our larger sample of 99 women with ED (52 AN, 47 BN), post-assessment time point was not at discharge of treatment, but defined as a 3-month treatment period, and even after this period, we found less decrease of sC in the clinical samples than in the healthy one. This result might be explained by the few full and partial remissions as they were only rarely achieved in the ED groups, and in the mixed mental control group, the effects of treatment were moderate at best (Schopf et al., 2023). Therefore, we assume that our findings can be interpreted as ongoing alterations of the psychophysiological stress regulation systems. To which extent this unchanged level of sC might represent a typical pattern of ED physiology or a premorbid vulnerability of ED (Het et al., 2020) that remains after treatment resulting in a lack of stress regulation has to be further investigated (Het et al., 2020; Munsch et al., 2021). Such premorbid physiological vulnerability has also been discussed in relation to early traumatic life experiences, e.g., maltreatment (Lo Sauro and colleagues, 2008; Monteleone et al., 2020; Meneguzzo et al., 2021), which leads to a heightened sensitivity to subjectively salient stimuli and increased anxious arousal, impacting the stress system and resulting in limited flexibility to adapt to challenging conditions or even blunted stress responses (Meneguzzo et al., 2021). In addition, such experiences have been associated with heightened cytokine levels, which seem to be linked with lower effects of psychotherapeutic interventions (Dalton et al., 2018). Besides this, dysregulated cortisol levels are not isolated hormonal changes in ED, but are part of a complex hormonal network system that might decelerate HPA normalization during treatment (Culbert et al., 2016). Therefore, sC changes can probably only be expected within an extended observation window (Het et al., 2020) and not after a 3-month treatment.

In addition, due to the comment of the editor regarding the utility of biomarkers in these clinical groups, we have added further information on page 28, it. Says now:

We investigated stress physiology in women with and without mental disorders and potential changes after treatment and in healthy women after a waiting period. As expected, we found indications that psychopathology in women with ED and mixed mental disorders translates into impairment of the HPA axis and the autonomic functioning in a resting condition that did not fully normalize over a 3-month treatment, although symptoms improved in the same period. Especially in severely underweight individuals, we assume that the short-term improvement may be dependent on weight recovery levels, and therefore, longer follow-up assessment periods are needed. Based on the results obtained in this study, it can be speculated that remaining dysregulated stress physiology after therapy might be related to prolonged recovery periods of the physiological system, also represented in low remission rates. Such physiological dysregulations are likely to impair the patient’s capacity to adequately react to daily challenges after in or outpatient treatment. If replicated, this finding underlines the need for ongoing aftercare support to prevent deterioration over time (Hegedüs et al., 2020). Such aftercare interventions could target stress system dysfunctions by training related to coping with psychological stress and by implementing breathing, meditation-oriented or biofeedback methods (Wendtz & Thayer, 2024) Mathersul et al., 2022). Nevertheless, a focus should also be put on improved aspects of emotion regulation, like alexithymia, as the capability to identify and name emotions or impulsivity is linked to a better interoception in people with ED (Martin et al., 2019; Meneguzzo et al., 2022). In addition, there is preliminary evidence that training gastric interoception might be more closely linked to ED pathology improvement (Tiemann et al., in press). Future research would profit from a harmonization of assessment methodology across studies and a merging of data in shared databases to allow for clearer comparisons of findings (Laufer et al., 2018).

***Editor:*** *The heterogeneity of the therapeutic interventions also warrants further attention. Although the manuscript acknowledges variability in setting, duration, and type of therapy, it does not sufficiently consider how these differences may have influenced the results. Addressing this issue in the interpretation—if not in the analyses—would help clarify the study’s conclusions.*

**Response:** Based on the comments of the editor and the reviewers, we now provide more detail on the influence of the therapeutic setting on the biological correlates of stress regulation and including comorbidity in the analyses. The information is provided on different pages in the manuscript. We added more information on our reasoning about the study design and limitations in our response to the reviewers, as we decided not to include the illness duration information, due to a high level of missing data. We comment on this point in the discussion section (p. 26). We further did not include covariates linked closely to the core symptoms of the disorders, such as psychological distress, which is an integral part of the psychopathology of our sample. Controlling for medication intake, physical activity, smoking habit, medication intake with effects on the HPA axis, use of contraceptives, patient type, comorbidity, and number of treatment sessions did not change the main findings (p.17-21).

Explanations are first provided in the method section, where it says now on page 14: “The following covariates were included for all analyses: physical activity, smoking habit, medication intake with effects on the HPA axis, and use of contraceptives. In addition, comorbidity (y/n) and patient type (out- or inpatient treatment/healthy) were considered for hypotheses focusing on comparisons exclusively between the clinical groups. Further, we considered the number of therapy sessions as a covariate whenever changes during treatment were at the center of our analyses including only clinical groups as in parts of hypotheses 2 and 4. Results concerning covariates are only reported if noteworthy, i.e., if the p-value of the association with the respective outcome was smaller than .01. Effect sizes are reported for all results.”

For hypothesis 1, p.18: “When disregarding healthy participants and controlling for the two additional covariates patient type, and comorbidity, mean values for sC were higher in the AN group compared to the combined BN/mixed group (a priori contrast, b=0.330, 95% CI=[0.115, 0.546], t(153)=3.03, p=.003, p_adj_=.005, d=0.55). Regarding the impact of covariates, mean values for sC and HF-HRV were lower in case of medication intake when comparing HC and clinical groups. In addition, HF-HRV mean values were also lower when comparing within clinical groups, only in the case of medication intake. In addition, outpatients exhibited higher values for sC than inpatients. All other covariates were never highly associated with any of the three outcomes sC, sAA, and HF-HRV in hypothesis 1.”

For hypothesis 2, p. 19: “Disregarding healthy participants and controlling for the three additional covariates patient type, number of therapy sessions, and comorbidity, the sC decrease in the combined AN/BN group was comparable to that in the mixed group (interaction "pre_post x AN/BN_vs_mixed", b=0.038, 95% CI=[–0.259, 0.183], t(136)=0.328, p=.743, p_adj_=1.0, d=0.09). … Regarding the impact of covariates on the change in outcomes between pre- and posttreatment, none were of importance for any of the three outcomes sC, sAA, and HF-HRV.”

For hypothesis 3, p.20: “Regarding the impact of covariates, none of them were highly associated with any of the outcomes sC, sAA, and HF-HRV at baseline, except that sC values were higher in outpatients than in inpatients in the combined BN/mixed groups.”

For hypothesis 4, p.21: “A noteworthy impact of covariates was only observed for the HF-HRV, which was associated with both the use of medication and of contraceptives. Thus HF-HRV was lower for medication intake than if no medication was taken and higher in patients using contraceptives than not using contraceptives.”

***Editor:*** *Furthermore, although sample size calculations are reported, high attrition rates may have affected the statistical power of longitudinal comparisons. A clearer discussion of dropout and how missing data were handled is needed.*

**Response:** We agree with the editor that attrition rates warrant consideration. Nevertheless, we found in our study that an equal number of women with or without mental disorders withdrew from participation before the study started, while 15% of all participating patients, compared to 1% of the women without mental disorders, dropped out from pre to posttreatment. We did not find any differences in age between those who stayed and those who prematurely terminated their participation.

A review by Fassino and colleagues reported clearly higher dropout rates than in our study, mounting up to 51% for inpatients and up to 79% for outpatient settings. But the authors found only a few differences between dropouts and ED patients who terminated their treatment. Only in terms of the tendency to binge purge and to emotionally overreact, dropouts differed from those who remained in the studies (Fassino et al., 2009).

To provide more information in our manuscript and describe our methodological approach to the attrition rates, we added now the following paragraph to the data analysis section on page 15: “Regarding missing values, the percentage of missing values in each variable involved in any of the analyses performed varied between 0 and 2.6, except for HF-HRV with a value of 11.4. Little's multivariate test for missing completely at random (MCAR) pattern did not reject the null hypothesis of MCAR (c^2^=65.5, df=67, p=0.53). When using multilevel models, the missing at random (MAR) pattern is expected to lead to unbiased estimates.”

***Editor:*** *Finally, several sections of the manuscript—particularly the introduction and discussion—would benefit from more concise and accessible writing to improve overall clarity.*

**Response:** We revised the introduction and discussion section according to the reviewers and hope that this has improved the clarity of our writing.

***Editor:*** *The authors are encouraged to revise the manuscript accordingly and to carefully consider the comments provided by the reviewers. The core idea of the study is valuable, and with these improvements, the manuscript could make a meaningful contribution to the field.*

**Response:** We thank you for the opportunity to revise the manuscript. We have considered all the comments, integrated information where needed, adapted the text and responded to each comment of the reviewers (see below).

**Review Comments to the Author**

***Reviewer #1****:*

***Reviewer:*** *The paper is well-written and methodologically clear. There is no specific concerns about this manuscript. However, I think it might be improved considering specific aspects:*

**Response:** We thank the reviewer for this feedback and the thoughtful evaluation of our manuscript.

***Reviewer:*** *The reduction in HF-HRV in AN/BN post-treatment is assumed to indicate normalization, but it is unclear whether this reflects improved autonomic flexibility or simply reduced overactivity*.

**Response:** We thank the reviewer for this comment. It is indeed difficult to assume whether changes of HF-HRV are the result of a sympathetic reduction induced by a lower stress level or rather a result of a high counterbalancing reaction in chronic stress conditions and therefore an overactivity. As we consider the HC group as characterized by high levels of mental health and therefore high functioning of the autonomic nervous system, we conclude that the approaching HF-HRV levels of the clinical samples towards the HC group levels rather represent a normalisation and therefore an increase of autonomic flexibility. We have now changed one part of the discussion section, and it says now on pages 24-25:

“Nevertheless, as expected, basal vagal tone decreased more between pre- and posttreatment in the AN/BN group than during waiting in healthy controls. The AN/BN group changes might indicate the beginning of a normalization process due to less food restriction and improved weight control, known to cause a desensitization of the physiological system and finally heightened vagal tone, even though full remission of ED-symptoms only rarely occurred in our study (Schopf et al., 2023). Similar changes have been found by Het and colleagues (2020). Vagal tone normalized during treatment and existing differences between ED and healthy volunteers before in-patient treatment did not exist anymore at discharge (Het et al., 2020). In contrast, data on only BN patients according to a recently published umbrella meta-analysis did not indicate such a normalization after treatment (Wang et al., 2025). In our study HRV values of clinical groups changed towards those of HC and we therefore assume that this corresponds with an improvement of parasympathetic activation either as a counter-regulation to still existing high levels of sympathetic activation or due to a reduction of stress causing an autonomic imbalance which might be restored in parallel to ED symptom improvement (Het et al.,2020; Miller et al., 2009). It is evident, that the mechanisms underlying vagal alterations are multifactorial (Wang et al., 2025) and therefore their understanding still needs further research. However, there is evidence that brain regions such as the prefrontal cortex and amygdala related to HRV functioning (including basal vagal tone) are also critical in regulating emotions and are positively influenced by psychotherapy contributing to symptom change (Wang et al.,2025) and probably to a higher autonomic flexibility. Future studies should investigate whether a continuous decrease of ED symptoms after psychotherapy in patients with ED might be related to ongoing improvements in physiological stress system, as has been observed in depressive and anxiety disorders after CBT (Smith et al., 2023).

As expected, the change in basal vagal tone in HC before and after the three-month waiting period was comparable to the change in the mixed group during treatment. Similar findings had been reported in a physically healthy sample with depression at post-psychotherapy (Kim et al., 2009), but not under other treatment conditions such as antidepressants (e.g., Neyer et al., 2021). In addition, previous studies on anxiety disorders showed consistent improvements of vagal tone (e.g. meta-analysis of Gonçalves et al., 2015), which let us assume that the biological defense system is not overactivated anymore after treatment.

Altogether, our findings support the distinct recovery abilities of the two physiological systems and underline the autonomic nervous system to be a faster-changing structure, whereas the HPA axis, being part of a complex hormonal system impacted at several levels by ED.

***Reviewer:*** *Cortisol levels (sC) did not significantly decrease, despite symptom improvement—potential explanations (e.g., chronic stress, metabolic adaptation, HPA-axis dysregulation) should be discussed.*

**Response:** As the reviewer correctly identified, sC levels did not decrease similarly in the clinical groups as in women without mental disorders. This may be due to different conditions, including the moderate improvement in core symptoms, which did not allow biological systems to recalibrate during the 3-month study duration. We now discuss these considerations in more detail on p. 23:

“Contrary to our expectations, sC levels decreased less in the clinical groups (AN, BN and mixed mental disorders) during therapy than during the waiting period in healthy women, whereas, as expected, the decrease of sAA was comparable between all clinical groups and the healthy sample. These findings might point to the long-lasting effect of ED pathologies on the physiological stress system even after weight normalization (Holsen et al., 2014), which are probably not reversed after a treatment duration of three months. Such treatment-resistant HPA activation patterns have also been reported by Het and colleagues (Het et al., 2020). They investigated a small sample of 13 young women with ED in comparison with a non-clinical sample and found at discharge from an in-patient treatment (at an average of 8.47 weeks SEM ± 1.8 (range: 2–58 weeks) after the first assessment) unchanged cortisol responses to a lab-based stress task. In our larger sample of 99 women with ED (52 AN, 47 BN), post-assessment time point was not at discharge of treatment, but defined as a 3-month treatment period, and even after this period, we found less decrease of sC in the clinical samples than in the healthy one. This result might be explained by the few full and partial remissions as they were only rarely achieved in the ED groups, and in the mixed mental control group, the effects of treatment were moderate at best (Schopf et al., 2023). Therefore, we assume that our findings can be interpreted as ongoing alterations of the psychophysiological stress regulation systems. To which extent this unchanged level of sC might represent a typical pattern of ED physiology or a premorbid vulnerability of ED (Het et al., 2020) that remains after treatment resulting in a lack of stress regulation has to be further investigated (Het et al., 2020; Munsch et al., 2021). Such premorbid physiological vulnerability has also been discussed in relation to early traumatic life experiences, e.g., maltreatment (Lo Sauro and colleagues, 2008; Monteleone et al., 2020; Meneguzzo et al., 2021). which leads to a heightened sensitivity to subjectively salient stimuli and increased anxious arousal, impacting the stress system and resulting in limited flexibility to adapt to challenging conditions or even blunted stress responses (Meneguzzo et al., 2021). In addition, such experiences have been associated with heightened cytokine levels, which seem to be linked with lower effects of psychotherapeutic interventions (Dalton et al., 2018). Besides this, dysregulated cortisol levels are not isolated hormonal changes in ED, but are part of a complex hormonal network system that might decelerate HPA normalization during treatment (Culbert et al., 2016). Therefore, sC changes can probably only be expected within an extended observation window (Het et al., 2020) and not after a 3-month treatment.

***Reviewer:*** *Please, consider as possible element that should be taken into serious consideration the effects of possible traumatic events that might characterized specific ED people (see* [*https://doi.org/10.1002/erv.2896*](https://urldefense.com/v3/__https:/doi.org/10.1002/erv.2896__;!!L4CEoEA!uSq9HAq_KWWdcTtUQyi2vwCxq6Ph5LKcZ7HvKf5IeDQs3cmBDugvweLGLNf9YcKH2cTw3D1ZR4Efxt4sLhDr$)*)*

**Response:** We agree that prior traumatic events could have influenced the responsivity of the stress system in ED and patients with other mental disorders. As we did not systematically assess the type, number and duration of traumatic events, we cannot include such data in our analysis, but comment on the potential effect in the discussion section on p 23:

“Such premorbid physiological vulnerability has also been discussed in relation to early traumatic life experiences, e.g., maltreatment (Lo Sauro and colleagues, 2008; Monteleone et al., 2020; Meneguzzo et al., 2021). which leads to a heightened sensitivity to subjectively salient stimuli and increased anxious arousal, impacting the stress system and resulting in limited flexibility to adapt to challenging conditions or even blunted stress responses (Meneguzzo et al., 2021).”

***Reviewer:*** *The study shows higher vagal tone in AN/BN and lower tone in anxiety/depression, but the mechanisms behind these differences are not fully explained.*

**Response:** We agree with the reviewer that we have not discussed this in detail. We have mentioned potential mechanisms related to ED-related behavior, however according to our knowledge and rediscussed in the recent umbrella meta-analysis of Wang et al. (2025), the exact mechanism of the most common alteration is still unclear. As mentioned above, we have integrated a sentence on the involved brain regions that are involved in autonomic functioning and emotion regulation on page 24:

“It is evident, that the mechanisms underlying vagal alterations are multifactorial (Wang et al., 2025) and therefore their understanding still needs further research. However, there is evidence that brain regions such as the prefrontal cortex and amygdala related to HRV functioning (including basal vagal tone) are also critical in regulating emotions and are positively influenced by psychotherapy contributing to symptom change (Wang et al.,2025) and probably to a higher autonomic flexibility…”

In our study, we discussed the expected larger decrease in vagal tone in the AN/BN versus the healthy group on p 19, and now additionally elaborate on the comparison of the mixed with the healthy group, which, in contrast to our expectations, was not significant on p. 20. It says: “These levels of basal vagal tone in ED might be the result of ED-related behaviors (food restriction, repeated binge and compensatory behavior) causing an overactivity of the vagal tone in ED patients and chronic stress levels related to the chronic activation of the fear-defense system in mixed disorders (e.g. Peyser et al., 2021; Watford et al., 2020; Messerli-Bürgy et al., 2010; Peschel et al., 2016).”

***Reviewer:*** *Does vagal overactivity in EDs result from chronic malnutrition, metabolic changes, or an adaptive parasympathetic dominance?*

**Response:** Thank you for these important questions. There are indeed different possible pathways that result in vagal overactivity. First, according to the allostatic load model (McEwen), chronic or repetitive stress can lead to an ongoing activity of the physiological stress system and lead to exhaustion, resulting in an allostatic load condition (McEwen, 1998) associated with persistent dysregulation of the autonomic nervous system and of the HPA axis at a resting state. Second, there is evidence that malnutrition, as found in AN and a history of extreme weight loss, both cause HPA dysregulation, which might be reversible after weight restoration (Chami et al., 2019). As the reviewer already pointed out, severe stress such as childhood trauma and childhood maltreatment, which are highly prevalent in ED, can cause HPA long-lasting dysregulation (Chami et al., 2019). Besides this, ongoing ED-specific symptoms such as self-starving, food restrictions, but also repeated binge eating and compensatory behavior, can result in an overactivity of the vagal tone. It might be assumed that constant activity of the stress system, like vagal overactivity, is followed by a heightened basal vagal tone according to the desensitization hypothesis and consequently limited flexibility to respond to emotional stimuli (Watford et al., 2020). We already included these ideas in the introduction section on p. 4 but reflect again on the mechanisms over time in the discussion section on p. 20.

***Reviewer:*** *BMI changes were not strongly correlated with stress physiology changes, which contradicts expectations. If BMI alone does not drive physiological normalization, what other factors (e.g., illness duration, stress exposure, psychological distress) play a role?*

**Response:** We agree that the association of BMI and physiology was not confirmed in the way we had expected. One of the explanations might be related to the existence of weight recovery. Previous studies on AN had revealed that only underweight recovery, HRV changes might be expected. In our sample, the remission rate was limited and therefore full weight recovery too, which might have impacted our results. We have added the relevance of weight recovery to the discussion section. It says now:

“The changes in physiological parameters during treatment could not be attributed to changes in BMI in AN, which might represent long-lasting physiological dysregulation in AN due to still ongoing reduced caloric intake within the 3 months of treatment and respective low weight gain (Schopf et al., 2023). Such a lack of association of BMI and HRV in AN has also been reported in patients without weight recovery (Mont et al., 2003: Paysal et al., 2022), and cardiac morphological remodeling has only been seen in those with weight recovery (Paysal et al., 2022). Further, even a short intensive re-nutrition and somatic stabilization intervention (mean length: 41 days) has not shown any cortisol changes in AN (Hemmingsen et al., 2022) and underlines the relevance of full recovery weight in terms of vagal recovery.

Further, we acknowledge that our study does not report information about the mechanisms behind the associations between disorder types and psychophysiology, which we measured. This would have implied running moderator analyses, which were beyond the scope of the current manuscript and would have required a larger sample size.

We further agree that we did not report much information about the role of covariates in our previous version of the manuscript, which we have now added. For methodological reasons, we did not include the illness duration information, as this information included too much missing data in our dataset. We comment on this point in the discussion section (p. 26). We also forewent including covariates that might be difficult to differentiate from the core symptoms of the disorders, such as psychological distress, which is an integral part of the psychopathology of our sample. Controlling for medication intake, physical activity, smoking habit, medication intake with effects on the HPA axis, use of contraceptives, patient type, comorbidity, and number of treatment sessions was done, but did not change the main findings (p.17-21).

***Reviewer:*** *The study highlights the need for aftercare, but lacks specific recommendations for improving physiological recovery in EDs.
- Could HRV biofeedback, vagal nerve stimulation, or stress regulation strategies help restore autonomic - Have you considered the difficulties that people with ED might have in the interoceptive awareness (see* [*https://doi.org/10.1007/s40519-022-01394-7)?*](https://urldefense.com/v3/__https:/doi.org/10.1007/s40519-022-01394-7)?__;!!L4CEoEA!uSq9HAq_KWWdcTtUQyi2vwCxq6Ph5LKcZ7HvKf5IeDQs3cmBDugvweLGLNf9YcKH2cTw3D1ZR4EfxmjPjeSP$)*balance?*

**Response:** We agree that there are specific intervention strategies that improve HRV in patients. Besides vagal stimulation, there are less invasive strategies that are often used. Several studies proved the impact of HRV biofeedback, but further slow breathing interventions, mindfulness-based but also meditation-based approaches have been considered besides physical exercising to improve vagal tone (Wendt & Thayer, 2024). Following the comments of the reviewer, we included such information on potential interventions and challenges in the Conclusion section; however, we remained more general, as state-of-the-art specific interventions were not the focus of this research program on p. 29.

***Reviewer:*** *Some sections are overly technical and dense, requiring clearer phrasing. Tables and figures should be formatted consistently for better readability.*

**Response:** We carefully worked on the language and phrasing of the whole manuscript and hope that these changes are satisfactory. We have further restructured table 1 and hope that this table is now easier to read.

**Reviewer #2:** Dear Authors,

***Reviewer:*** *First, I would like to congratulate you on the development of an original and relevant study that contributes to the understanding of stress physiology in women with eating disorders (EDs) and mixed disorders. Below, I detail substantive and specific observations that could further strengthen the manuscript:*

**Response:** We thank the reviewer for this positive comment and appreciate his feedback. We have worked on each of them and have adapted the text accordingly.

***Reviewer:*** *Heterogeneity of treatments (main interpretive limitation): Although treatments followed national clinical guidelines, their duration, modality (inpatient/ambulatory), and number of sessions varied widely. This heterogeneity is recognized as a covariate, but remains a factor limiting causal attribution between treatment and physiological changes. I recommend further analysis of how this variability may have differentially influenced biomarkers. In addition to considering exploratory sub-analyses by treatment modality (e.g., outpatient vs. inpatient).*

**Response:** We thank the reviewer for this comment. We have recalculated our analyses and have now included additional covariates. These encompass physical activity, smoking habit, medication intake, use of contraceptives, comorbidity, patient type, and number of psychotherapy sessions provided. The covariates are all listed in the data analysis section, but also in the hypothesis section at the end of the introduction.

Concerning exploratory sub-analyses as raised by the reviewer, we see that they would be an interesting research topic. However, we believe that this would be a research topic and would go beyond the scope of this manuscript. Also, this kind of analysis (i.e., moderation models) requires larger sample sizes, and we do not have enough power in our sample to conduct moderation analysis.

However, we agree that the results on physiological changes in relation to BMI during treatment need to be better explained and discussed. We have therefore added several sections to the discussion.

In terms of physiological changes within different groups, we have added on p. 25:

“We did not find any association of sC at rest and BMI in AN or the combined BN and mixed groups, nor did we find a relation of sAA levels or HF-HRV with BMI in the clinical groups, These findings do not confirm what has been found previously in healthy individuals (e.g. meta-analysis of Koenig et al., 2014). However, BMI clearly being different in AN from BN or mixed group, we did not find an association with levels of basal vagal tone and therefore assume that HRV in this case rather represents the underlying emotion regulation difficulties which are related to higher medial prefrontal cortex and amygdala functional connectivity (Sakaki et al., 2016; Jennings et al., 2016) and to less emotional wellbeing (Mather & Thayer, 2018). However, physical condition represented by BMI does not show this association with vagal tone in our study, and is rather linked to the symptomatology of the different pathologies in our sample (see hypothesis 1).”

About the BMI changes being related to physiological changes in AN: We have added the following on page 25:

“Such a lack of association of BMI and HRV in AN has also been reported in patients without weight recovery (Mont et al., 2003: Paysal et al., 2022), and cardiac morphological remodeling has only been seen in those with weight recovery (Paysal et al., 2022). Further, even a short intensive re-nutrition and somatic stabilization intervention (mean length: 41 days) has not shown any cortisol changes in AN (Hemmingsen et al., 2022) and underlines the relevance of full recovery weight in terms of vagal recovery.“

***Reviewer:*** *Salivary alpha amylase (sAA): expected elevation of sAA in BN and mixed group, but data did not confirm this. could the time of collection (late) or experimental design have influenced this? Discuss how the diurnal sAA curve or anticipatory response to the experimental context might have flattened the expected differences. It may be more informative to use it as an index of acute response to the experimental stressor, rather than measuring basal levels under poorly standardized conditions*.

**Response:** We agree with the reviewer that the assessment time point during the overall study cannot be labeled as a standard resting-state assessment. The young women had subscribed to participate in an experimental trial with two assessment time points before and after treatment as usual or a waiting period of equal length for women without mental disorders. The conditions were highly standardized following a rigorous standardized operation protocol, which has been outlined in this manuscript and, e.g., in Humbel et al. (2018) or Munsch et al. (2021). We exclusively used baseline measures before the experimental paradigm for this study. Therefore, our findings cannot be interpreted to reflect an index of acute response to the experimental stressor. We have now defined assessment time points used in this study more clearly on p. 9 in the methods section.

We agree that the influence of the participation per se cannot be ruled out to be an influencing factor on biological assessments in this study. In this line, we previously showed in Wyssen et al. (2016), a general anticipatory arousal to this experimental setup and a subsequent habituation to the experimental manipulation in a different and healthy sample of young women and a general decline of biological measures throughout the experimental laboratory paradigm applied in this sample in a previous publication of our group (Munsch et al., 2021). Nevertheless, in this study, we used the two measures of biological samples before the experimental task, one 5 minutes after arrival at the laboratory and one 30 minutes after arrival, after installation of the equipment, which should have buffered the anticipatory effect on stress hormones (p. 9, 11). Therefore, we calculated mean values of the two assessment time points for sC and sAA at rest. We now comment on these points in the limitation section on p. 22. It says: “It further has to be considered that the physiological parameters assessed are known to vary depending on the type of stress exposure at or just before assessment (e.g. acute daily stressors or currently high levels due to the lab condition). Therefore, repeated sampling of sC and sAA was used and calculations of mean levels were considered to better define resting levels, and the assessment period (only between 2 pm and 4.30 pm (Krieger et al., 1975) was set to be limited. Nevertheless, the measures were taken from women anticipating a laboratory experiment on thin ideals and eating behavior, but physiological stress measures remained low in our sample.”

Further, the reviewer asked to comment on the potential impact of diurnal sAA levels on physiological measures in the lab session. Due to the diurnal rhythm of physiological measures, the study protocol considered a strict period for all the testing. That means, all testing in the lab has taken place between 2 and 4.30 p.m. at pre- and post-assessment, where we expected less changing sAA levels (Rohleder & Nater, 2009; Adam et al., 2011).

***Reviewer:*** *Salivary cortisol (sC): In the discussion, consider the influence of the circadian rhythm of cortisol (collection between 14:00-16:30 limits interpretation due to the descending phase of the diurnal curve). In addition, it should be mentioned as a limitation that cortisol on awakening (CAR) was not considered as a more robust marker of HPA axis activation in these clinical pictures.*

**Response:** Thank you for this comment. We agree that the time point of the laboratory assessment of biological measures was aligned with the circadian rhythm of the HPA-axis and the ANS system and according to the gold standard, all lab sessions took place in the afternoon, where physiological measures are less influenced by the circadian rhythm. However, we have now included a sentence in the limitation section on this point. It says now: “Nevertheless, the measures were taken from women anticipating a laboratory experiment on thin ideals and eating behavior, but physiological stress measures remained low in our sample. Besides this, there might be other potential confounders that impact physiological stress responses, e.g., illness duration, circadian rhythm, which could not be considered in our statistical analyses.

The assessment of cortisol awakening response would demand a different study approach, asking participants to collect saliva at home in the morning after awakening and repeatedly within the following 60 minutes (Stadler et al.2022). The CAR represents a relatively distinct feature within the circadian rhythmicity (Stadler et al.), which is not closely related to earlier or later phases and is influenced by light exposure and time of awakening but further by anticipation of diurnal stress. In this study design, the focus was on potential changes in physiological measures, symptoms, but also underlying mechanisms of eating disorders (e.g., thought shape fusion), which were experimentally evaluated. Due to this focus, the assessment of CAR was not planned. But we agree that CAR is a robust marker, although the literature shows that a lack of change in cortisol is also found in CAR data on patients with eating disorders. For instance, in a study of Wild et al. (2014) mean CAR of patients with severe symptoms was still higher at post-assessment than that of those with less severe symptoms.

***Reviewer:*** *Underutilized clinical and psychopathological variables: they did not include an analysis by duration of EDs or specific comorbidities, which could modulate the physiological response. If these data are available, it is recommended that they be incorporated into the analysis.*

**Response:** We have received a similar comment from Reviewer One and have mentioned this above. The main goal of the study and the sample size determined our analyses. We understand that more information on the influence of the covariates is needed, and we now report our new results (p 17-20). Besides physical activity, smoking habit, and medication intake with effects on the HPA axis, and use of contraceptives, patient type (in-/outpatient), we now include two more covariates: comorbidity rate and number of psychotherapy sessions. However, the suggested variable “duration of ED” could not be considered as covariate, as data was not available (e.g. many missing data, not reliable report of patients).

It says now in the hypotheses: “More precisely, at pretreatment, **we first** expected elevated sC mean values in AN compared to the remaining groups of BN, mixed, and healthy controls, …. For this hypothesis we included the following four covariates: physical activity, smoking habit, medication intake with effects on the HPA axis, and use of contraceptives. Disregarding healthy controls, we further expected elevated sC mean values in AN compared to the clinical groups of BN and mixed... For this hypothesis, we included the same covariates as before, plus the following three covariates, which could not be included in the first hypothesis since they were completely confounded with the healthy control group: patient type (in-/outpatient), number of therapy sessions, and comorbidity.

**Second**, we expected comparable changes during treatment (or after waiting for healthy controls) in stress physiology in clinical groups compared to healthy controls ..For this hypothesis, physical activity, smoking habit, medication intake with effects on the HPA axis, and use of contraceptives were included as covariates. Disregarding healthy controls, we further expected a higher decrease in sC, sAA and for basal vagal tone in AN and BN than in the mixed group, controlling for in- and outpatient setting, number of sessions and comorbidity. For this hypothesis, the aforementioned 4 covariates, plus patient type (in-/outpatient), number of therapy sessions, and comorbidity, were included as covariates.

**Third**, at pretreatment, we expected for sC … For this hypothesis, physical activity, smoking habit, medication intake with effects on the HPA axis, and use of contraceptives, plus patient type (in-/outpatient) and comorbidity, were included as covariates. **Finally**, fourth, we expected a temporal association between BMI and sC, sAA, and basal vagal tone during treatment in the AN group, including physical activity, smoking habit, medication intake with effects on the HPA axis, and use of contraceptives, plus patient type (in-/outpatient), number of therapy sessions and comorbidity as covariates.

***Reviewer:*** *Absence of associations between BMI and biomarkers: The expected correlations between BMI and vagal tone were not observed in AN. This could reflect limitations of HRV as an index of emotional regulation in severely malnourished individuals. I recommend considering whether physiological dysregulation in AN may be less sensitive to short-term weight variations.*

**Response:** We agree with the reviewer, that the lack of full weight recovery might have influenced our results. We have now added a section that says:

“Such a lack of association of BMI and HRV in AN has also been reported in patients without weight recovery (Mont et al., 2003: Paysal et al., 2022), and cardiac morphological remodeling has only been seen in those with weight recovery (Paysal et al., 2022). Further, even a short intensive re-nutrition and somatic stabilization intervention (mean length: 41 days) has not shown any cortisol changes in AN (Hemmingsen et al., 2022) and underlines the relevance of full recovery weight in terms of vagal recovery.”

***Reviewer:*** *Also, discuss whether duration of disorder or chronicity (used as a covariate) might have influenced the physiological response.*

**Response:** We agree that the duration of illness might be a proxy for chronicity and would be a preferential covariate. Unfortunately, this variable included many missing values, and we had to forego including this information in our study. We comment on this problem in our limitations section on p 26. Instead, we included the comorbidity status and psychotherapy sessions as covariates in the models and report on the extent of the impact of all covariates whenever it is worth mentioning on pages 17-20.

***Reviewer:*** *Minor comments: In methodology clarify the use of repeated measures: was the mean of the two saliva samples used for sC and sAA? In conclusion, the manuscript has potential, but needs some adjustments and clarifications to reach an even higher level of interpretive rigor.*

**Response:** 89e calculated mean values of sAA and sC at rest. We have added this information. It says now: “Therefore, repeated sampling of sC and sAA was used and calculations of mean levels were considered to better define resting levels, and the assessment period (only between 2 pm and 4.30 pm (Krieger et al., 1975) was set to be limited. Nevertheless, the measures were taken from women anticipating a laboratory experiment on thin ideals and eating behavior, but physiological stress measures remained low in our sample.”
We thank the reviewer for this positive feedback and his evaluation. We have made the changes and responded to each of the comments.

***Reviewer:*** *In its current form, the findings suggest some dissociation between clinical improvement and physiological modulation, which should be further discussed as an indicator of:
- Persistent dysfunction of stress systems despite symptomatic improvements,
- Or, of methodological limitations in capturing subtle physiological changes over short periods of time.*

**Response:** We thank the reviewer for this precise summary of the potential interpretation and limitations of our findings and hope to have addressed both points now in our revised discussion (pp. 19-21), limitations (p. 22), and conclusion sections on p. 23.

**Old References ! please replace with new ones**

Adam, E. K., Hoyt, L. T., & Granger, D. A. (2011). Diurnal alpha amylase patterns in adolescents: Associations with puberty and momentary mood states. Biological psychology, 88(2-3), 170-173.

Chami, R., Monteleone, A. M., Treasure, J., & Monteleone, P. (2019). Stress hormones and eating disorders. *Molecular and Cellular Endocrinology*, *497*, 110349. <https://doi.org/10.1016/j.mce.2018.12.009>

Culbert, K. M., Racine, S. E., & Klump, K. L. (2016). Hormonal factors and disturbances in eating disorders. *Current Psychiatry Reports, 18*(7), Article 65. <https://doi.org/10.1007/s11920-016-0701-6>

Fassino, S., Pierò, A., Tomba, E., & Abbate-Daga, G. (2009). Factors associated with dropout from treatment for eating disorders: A comprehensive literature review. *BMC Psychiatry, 9*, Article 67. https://doi.org/10.1186/1471-244X-9-67

Gonçalves, R., Rodrigues, H., Novaes, F., Arbol, J., Volchan, E., Coutinho, E. S. F., Figueira, I., & Ventura, P. (2015). Listening to the heart: A meta-analysis of cognitive behavior therapy impact on the heart rate of patients with anxiety disorders. *Journal of Affective Disorders, 172*, 231–240. <https://doi.org/10.1016/j.jad.2014.09.058>

Hemmingsen, S. D., Jensen, N. A., Larsen, P. V., Sjögren, J. M., Lichtenstein, M. B., & Støving, R. K. (2022). Cortisol, depression, and anxiety levels before and after short‑term intensive nutritional stabilization in patients with severe anorexia nervosa. *Frontiers in Psychiatry, 13*, Article 939225. https://doi.org/10.3389/fpsyt.2022.939225

Het, S., Vocks, S., Wolf, J., Herpertz, S., & Wolf, O. (2020). Treatment‑resistant blunted HPA activity, but reversible cardiovascular stress reactivity in young women with eating disorders. Frontiers in Psychiatry, 11, Article 726. https://doi.org/10.3389/fpsyt.2020.00726

Holsen, L. M., Lawson, E. A., Christensen, K., Klibanski, A., & Goldstein, J. M. (2014). Abnormal relationships between the neural response to high- and low-calorie foods and endogenous acylated ghrelin in women with active and weight-recovered anorexia nervosa. *Psychiatry Research: Neuroimaging, 223*(2), 94–103. https://doi.org/10.1016/j.pscychresns.2014.04.015

Humbel, N., Messerli-Bürgy, N., Schuck, K., Wyssen, A., Garcia-Burgos, D., Biedert, E., Lennertz, J., Meyer, A. H., Whinyates, K., Isenschmid, B., Milos, G., Trier, S., Adolph, D., Cwik, J., Margraf, J., Assion, H.-J., Teismann, T., Ueberberg, B., Juckel, G., … Munsch, S. (2018). Self-reported emotion regulation difficulties are associated with mood but not with the biological stress response to thin ideal exposure. *PloS One*, *13*(6), e0199769. https://doi.org/10.1371/journal.pone.0199769

Kim, W., Lim, S., Chung, E., & Woo, J. (2009). The effect of cognitive behavior therapy-based psychotherapy applied in a forest environment on physiological changes and remission of major depressive disorder. *Psychiatry Investigation, 6*(4), 245–254. <https://doi.org/10.4306/pi.2009.6.4.245>

Martin, E., Dourish, C. T., Rotshtein, P., Spetter, M. S., & Higgs, S. (2019). Interoception and disordered eating: A systematic review. *Neuroscience and Biobehavioral Reviews, 107*, 166–191. https://doi.org/10.1016/j.neubiorev.2019.08.020

McEWEN, B. S. (1998). Stress, Adaptation, and Disease: Allostasis and Allostatic Load. *Annals of the New York Academy of Sciences*, *840*(1), 33–44. <https://doi.org/10.1111/j.1749-6632.1998.tb09546.x>

Meneguzzo, P., Mancini, C., Ormitti, A., Bonello, E., & Todisco, P. (2022). Time evaluation and its accuracy in eating disorders: Differences in relation to interoceptive awareness. Eating and Weight Disorders – Studies on Anorexia, Bulimia and Obesity, 27(7), 2551–2560. <https://doi.org/10.1007/s40519-022-01394-7>

Messerli-Bürgy, N., Engesser, C., Lemmenmeier, E., Steptoe, A., & Laederach-Hofmann, K. (2010). Cardiovascular stress reactivity and recovery in bulimia nervosa and binge eating disorder. *International Journal of Psychophysiology*, *78*(2), 163–168. <https://doi.org/10.1016/j.ijpsycho.2010.07.005>

Miller, S. P., Erickson, S. J., Branom, C., & Steiner, H. (2009). Habitual response to stress in recovering adolescent anorexic patients. *Child Psychiatry and Human Development, 40*(1), 43–54. https://doi.org/10.1007/s10578-008-0112-y

Mont, L., Castro, J., Herreros, B., San José, E., Pérez, J. M., Vidal, B., Navarro, C., Esteban, C., Pomar, E., Berruezo, A., García-Honduvilla, N., & Brugada, P. (2003). Reversibility of cardiac abnormalities in adolescents with anorexia nervosa after weight recovery. *Journal of the American Academy of Child & Adolescent Psychiatry, 42*(7), 808–813. https://doi.org/10.1097/01.CHI.0000046867.56865.EB

Munsch, S., Messerli-Bürgy, N., Meyer, A. H., Humbel, N., Schopf, K., Wyssen, A., Forrer, F., Biedert, E., Lennertz, J., Trier, S., Isenschmid, B., Milos, G., Claussen, M., Whinyates, K., Adolph, D., Margraf, J., Assion, H.-J., Teismann, T., Ueberberg, B., … Schneider, S. (2021). Consequences of exposure to the thin ideal in mass media depend on moderators in young women: An experimental study. *Journal of Abnormal Psychology*, *130*(5), 498–511. https://doi.org/10.1037/abn0000676

Neyer, S., Witthöft, M., Cropley, M., Pawelzik, M., Lugo, R. G., & Sütterlin, S. (2021). Reduction of depressive symptoms during inpatient treatment is not associated with changes in heart rate variability. *PLOS ONE*, *16*(3), e0248686. https://doi.org/10.1371/journal.pone.0248686

Paysal, J., Thireau, J., Terral, D., Rochette, E., Obert, P., Merlin, E., & Nottin, S. (2022). Cardiac remodeling and its determinants in anorexia nervosa adolescents: Impact of weight recovery. *Children, 9*(4), 458. https://doi.org/10.3390/children9040458

Peschel, S. K. V., Feeling, N. R., Vögele, C., Kaess, M., Thayer, J. F., & Koenig, J. (2016). A Meta-analysis on Resting State High-frequency Heart Rate Variability in Bulimia Nervosa. *European Eating Disorders Review*, *24*(5), 355–365. https://doi.org/10.1002/erv.2454

Peyser, D., Scolnick, B., Hildebrandt, T., & Taylor, J. A. (2021a). Heart rate variability as a biomarker for anorexia nervosa: A review. *European Eating Disorders Review*, *29*(1), 20–31. https://doi.org/10.1002/erv.2791

Peyser, D., Scolnick, B., Hildebrandt, T., & Taylor, J. A. (2021b). Heart rate variability as a biomarker for anorexia nervosa: A review. *European Eating Disorders Review*, *29*(1), 20–31. https://doi.org/10.1002/erv.2791

Rohleder, N., & Nater, U. M. (2009). Determinants of salivary α-amylase in humans and methodological considerations. Psychoneuroendocrinology, *34*(4), 469-485.

Schopf, K., Schneider, S., Meyer, A. H., Lennertz, J., Humbel, N., Bürgy, N.-M., Wyssen, A., Biedert, E., Isenschmid, B., Milos, G., Claussen, M., Trier, S., Whinyates, K., Adolph, D., Teismann, T., Margraf, J., Assion, H.-J., Überberg, B., Juckel, G., … Munsch, S. (2023). Eating disorder treatment in routine clinical care: A descriptive study examining treatment characteristics and short-term treatment outcomes among patients with anorexia nervosa and bulimia nervosa in Germany and Switzerland. *PLOS ONE*, *18*(6), e0280402. https://doi.org/10.1371/journal.pone.0280402

Smith, K. E., Mason, T. B., Crosby, R. D., Cao, L., Leonard, R. C., Wetterneck, C. T., Smith, B. E. R., Farrell, N. R., Riemann, B. C., Wonderlich, S. A., & Moessner, M. (2019). A comparative network analysis of eating disorder psychopathology and co-occurring depression and anxiety symptoms before and after treatment. *Psychological Medicine*, *49*(2), 314–324. <https://doi.org/10.1017/S0033291718000867>

Smith, O. R. F., Aarø, L. E., & Knapstad, M. (2023). The Importance of Symptom Reduction for Functional Improvement after Cognitive Behavioral Therapy for Anxiety and Depression: A Causal Mediation Analysis. Psychotherapy and Psychosomatics, 92(3), 193–202. https://doi.org/10.1159/000530650

Stalder, T., Lupien, S. J., Kudielka, B. M., Adam, E. K., Pruessner, J. C., Wüst, S., Dockray, S., Smyth, N., Evans, P., Kirschbaum, C., Miller, R., Wetherell, M. A., Finke, J. B., Klucken, T., & Clow, A. (2022). Evaluation and update of the expert consensus guidelines for the assessment of the cortisol awakening response (CAR). *Psychoneuroendocrinology, 146*, Article 105946. https://doi.org/10.1016/j.psyneuen.2022.105946

Tiemann, A., Ortmann, J., Rubo, M., Meyer, A. H., Munsch, S., Vögele, C., & van Dyck, Z. (2025). The relevance of cardiac and gastric interoception for disordered eating behavior. Journal of Eating Disorders, 13, Article 114. https://doi.org/10.1186/s40337-025-01284-0

Wang, Z., Zou, Y., Liu, J., Peng, W., Li, M., Zhang, X., … Zou, Z. (2025). Heart rate variability in mental disorders: An umbrella review of meta-analyses. *Translational Psychiatry, 15*, Article 104. <https://doi.org/10.1038/s41398-025-03339-x>

Watford, T. S., Braden, A., & O’Brien, W. H. (2020). Resting state heart rate variability in clinical and subthreshold disordered eating: A meta-analysis. *International Journal of Eating Disorders*, *53*(7), 1021–1033. <https://doi.org/10.1002/eat.23287>

Wendt, J., & Thayer, J. F. (2024). Heart rate variability in mental health and psychotherapy. *Integrating Psychotherapy and Psychophysiology: Theory, Assessment, and Practice; Steffen, P., Moss, D., Eds*, 75-100.

Wild, B., Wesche, D., Schultz, J. H., Stroe-Kunold, E., Herzog, W., Rudofsky, G., Maser-Gluth, C., & Friederich, H. C. (2014). Trajectories of the cortisol awakening responses during weight gain in anorexia nervosa patients with severe and less severe symptoms. *International Journal of Psychophysiology, 94*(3), 272–277. <https://doi.org/10.1016/j.ijpsycho.2014.09.010>

Wyssen, A., Coelho, J. S., Wilhelm, P., Zimmermann, G., & Munsch, S. (2016). Thought-shape fusion in young healthy females appears after vivid imagination of thin ideals. *Journal of Behavior Therapy and Experimental Psychiatry*, *52*, 75–82. https://doi.org/10.1016/j.jbtep.2016.03.010
